# Ibuprofen for Ductus Arteriosus Months after Birth

**DOI:** 10.1155/2016/2659389

**Published:** 2016-06-14

**Authors:** Odile Frauenfelder, Ingrid M. van Beynum, Irwin K. M. Reiss, Sinno H. P. Simons

**Affiliations:** ^1^Division of Neonatology, Department of Pediatrics, Erasmus MC-Sophia Children's Hospital, Wytemaweg 80, 3015 CN Rotterdam, Netherlands; ^2^Division of Pediatric Cardiology, Department of Pediatrics, Erasmus MC-Sophia Children's Hospital, Wytemaweg 80, 3015 CN Rotterdam, Netherlands

## Abstract

Ibuprofen is a well-known agent used to treat patent ductus arteriosus in preterm neonates in the first days of life. In the current case report we illustrate the potential use of ibuprofen in two preterm neonates 60 and 88 days after birth, respectively. To our knowledge, this is the first report on the effects of ibuprofen on patent ductus arteriosus in preterm newborns after months of life. These cases suggest that the ductus arteriosus does not become refractory for ibuprofen after the first days of life. Late closure of the duct with ibuprofen might still improve the cardiorespiratory condition and prevent infants from surgical closure. Controlled trials are necessary to further study these findings.

## 1. Introduction

In preterm neonates, the ductus arteriosus often remains open after birth. Pharmacologically, this patent ductus arteriosus (PDA) can be closed with Non-Steroidal Anti-Inflammatory Drugs (NSAIDs), such as ibuprofen and indomethacin [1]. There is an ongoing international discussion about how and when PDA in preterm neonates should be treated. Prophylactic treatment with ibuprofen is unnecessary, because the ductus arteriosus might close spontaneously in a considerable part of patients and ibuprofen is related to side effects in newborns [2]. Whether preterm newborns with a hemodynamically significant PDA (hsPDA) need early (first postnatal days) or late (within first 2 weeks) treatment is unknown. Ibuprofen dosages need to be increased with older postnatal ages because of a faster clearance [3]. However, ibuprofen therapy is hypothesized to be less effective at older postnatal ages because the ductus arteriosus might become therapy resistant [4]. As PDA and reopening of the ductus arteriosus are also related to neonatal sepsis, therapy resistance might be due to increased inflammatory processes in the neonatal duct. Persistence of the duct is also related to hypoxia/hyperoxia, nitric oxide, cytokines, and hormones. Although animal data suggest postnatal remodeling and maturation the exact mechanism and pharmacodynamic differences need to be unraveled [5]. Clinical data are lacking about ibuprofen's efficacy on PDA after the first weeks of life. We present two cases of postponed PDA treatment with ibuprofen in preterm neonates.

## 2. Case 1

Case 1 was a preterm boy born by caesarian section because of maternal preeclampsia after 30 weeks and 5 days, after maternal corticosteroids, with a birth weight of 1280 grams and Apgar scores of 9 and 9 after 1 and 5 minutes, respectively. He was treated twice with surfactant because of respiratory distress syndrome and subsequently treated with nasal CPAP.

The first cardiac ultrasound was made at the 9th day of life and showed a large hemodynamically significant PDA. The PDA was not treated, because of a* Staphylococcus aureus* sepsis at that time. Unfortunately, the bacteremia persisted under antibiotic treatment and an intracardiac thrombus was found. The patient was treated with low molecular weight heparin and antibiotics for more than 6 weeks. During this period, several cardiac ultrasounds were made and all showed the PDA. The patient was cardiorespiratory stabile while treated with diuretics. CPAP was weaned successfully.

At the age of 58 days, weeks after recovery from the bacteremia, the patient's respiratory condition worsened and he showed increased oedema. He needed more respiratory support; Optiflow support was started with oxygen therapy up to 30%. Cardiac ultrasound showed a hemodynamically significant PDA (see Table 1). Intravenous ibuprofen was started at 20 mg/kg on the first day of treatment at a postnatal age of 60 days and maintained at 10 mg/kg per day. After 3 days of ibuprofen treatment, narrowing of the PDA was seen on echocardiography and we decided to continue treatment for another 3 days. At the 67th postnatal day, the concomitant murmur had disappeared and the ductus arteriosus was almost completely closed on ultrasound. The respiratory condition improved and the patient was transported to another hospital at the age of 72 days.

## 3. Case 2

Case 2 was a preterm born girl, first of twins, born after 25 weeks of gestation with a birthweight of 645 grams and Apgar scores of 1, 6, and 8 after 1, 5, and 10 minutes, respectively. She received surfactant once via an INSURE (endotracheal intubation, surfactant replacement therapy, and fast extubation) method. She needed noninvasive ventilation from postnatal day 5 until day 43.

A hsPDA was found (PDA, 1.9 mm, PDA/LPA ratio, 1.0) at postnatal day 6 and treated with intravenous ibuprofen during the next 6 consecutive days (dosages: 10-5-5-10-5-5 mg/kg). Afterwards, the ductus unfortunately turned out to be still open and diuretics were started to reduce pulmonary fluid overload. Because the cardiopulmonary condition stabilized surgical ductus ligation was not performed. The patient developed a severe BPD with 36% oxygen and 6 liters/minute Optiflow at postmenstrual age of 36 weeks.

Because her cardiopulmonary condition worsened at the age of 88 days (PMA 37 + 4 weeks) a new cardiac ultrasound was performed. A hsPDA was still present. We decided to treat the patient again with ibuprofen during a total of 6 days (oral ibuprofen, dosage: 20-10-10-10-10-10 mg/kg). Cardiac ultrasound (see Table 1) again reduced the size of the PDA, but the effect was less explicit. Clinically, the cardiorespiratory support could be gradually reduced (Optiflow 5 L/min, 35% oxygen before start to nasal cannula, 1 L/min, 25% oxygen within 2 weeks).

## 4. Discussion

**Figure 1:**
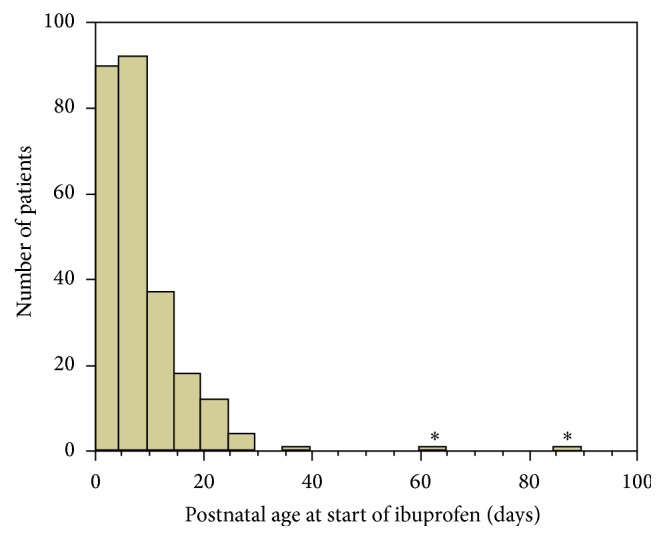
Numbers of patients for postnatal age (in days) at start of ibuprofen PDA therapy. Total *N* = 256 patients who received ibuprofen for PDA between October 2011 and November 2015 in our NICU. *∗* represents the current cases, illustrating the extremely late treatment of the current patient.

To our knowledge, these are the first reported cases of ibuprofen therapy for PDA in premature infants treated after postnatal ages of 2-3 months. In most patients described in literature PDA treatment is started within the 72 hours of life [6] or within the first 2 weeks of life in hospitals that wait for spontaneous ductus closure first [7].  Figure 1 shows the postnatal ages of patients treated in our hospital with ibuprofen during the last years.

The first patient had a relatively old gestational age compared to other preterm neonates treated with ibuprofen reported in the literature. In patients born after 30 weeks of gestation, the ductus arteriosus often closes spontaneously. It is unlikely, however, that the detected effect of the ductus was a spontaneous phenomenon. Initially, in the first patient, the patent duct was not treated because of ongoing sepsis but was open on repetitive ultrasounds. Weeks after appropriate treatment of the sepsis, the duct was large and became hemodynamically important. The ibuprofen dosages used are relatively high, compared to those advised during the first days of life. Ibuprofen clearance increases during the first weeks of life and the increased dosages seem to be adequate [3, 6]. Pharmacokinetic/pharmacodynamic data of ibuprofen use in newborns treated for PDA after the first week are missing, and the sufficiency of our dosing strategy is unclear. The effect of ibuprofen in the second patient was less clear on ultrasound, but the patient's clinical condition improved. It underlines that these results should be interpreted with caution because they only describe two cases. Well-designed clinical controlled trials are necessary to further study the effects of ibuprofen at older ages.

In conclusion, our case reports suggest that ibuprofen might still be effective for PDA closure in preterm neonates even after 60 days of life. As a consequence, it should be taken into account as a potential treatment option in case of prolonged hsPDA.

## Figures and Tables

**Table 1:** Ultrasound results of Case 1 and Case 2 before, during, and after treatment with ibuprofen.

PNA (days)	PDA (mm)	PDA/LPA (ratio)	LA/Ao (ratio)	LVID*z*-score	Li-Re shunt (*V*max, m/s)	Conclusion PDA
Case 1						

Day 58	3.1	0.76	1.8	+2.3	2.1	Large

Ibuprofen postnatal days 60, 61, and 62						
Day 63	2.1	0.51	1.6	+1.6	4.0	Moderate

Ibuprofen postnatal days 63, 64, and 65						
Day 67	0.8	0.20	1.6	+0.87	4.1	Small

Case 2						

Day 87	2.3	0.52	1.6	—	2.9	Moderate

Ibuprofen postnatal days 88, 89, and 90						
Day 91	1.5	0.34	1.6	+3.3	3.9	Small/moderate

Ibuprofen postnatal days 91, 92, and 93						
Day 94	1.4	0.30	1.5	+1.9	3.8	Small

PNA: postnatal age; PDA: patent ductus arteriosus; LPA: left pulmonary artery; LA: left atrium; Ao: aorta; LVID: left ventricle internal diameter.
